# An Assessment of the Prevalence of Dental Caries, Oral Hygiene Status, Deft Index, and Oral Hygiene Habits Among Children With Special Healthcare Needs

**DOI:** 10.7759/cureus.42416

**Published:** 2023-07-25

**Authors:** Shikha Shalini, Swati Sharma, Abhishek Anand, Sultan A Almalki, Arpan Biswas, Mridul Sharma, Tarun Sihag, Akshaya Ojha, Yogesh Garg, Kapil Paiwal

**Affiliations:** 1 Department of Orthodontics and Dentofacial Orthopaedics, Smitam Arogyam Dental Centre, Patna, IND; 2 Department of Pedodontics and Preventive Dentistry, Dental College RIMS, Ranchi, IND; 3 Department of Pedodontics and Preventive Dentistry, Netaji Subhas Medical College and Hospital, Patna, IND; 4 Department of Preventive Dental Sciences, Prince Sattam Bin Abdul Aziz University, Al Kharj, SAU; 5 Department of Public Health Dentistry, Vyas Dental College and Hospital, Jodhpur, IND; 6 Faculty of Dentistry, McGill University, Montreal, CAN; 7 Department of Pedodontics and Preventive Dentistry, Geetanjali Dental College, Udaipur, IND; 8 Department of Pedodontics and Preventive Dentistry, Private Practice, Jammu, IND; 9 Department of Public Health Dentistry, JCD Dental College, Sirsa, IND; 10 Department of Oral and Maxillofacial Pathology, Daswani Dental College and Research Center, Kota, IND

**Keywords:** dental caries, habits, children, oral hygiene habits, deft index

## Abstract

Background and objective

Children with special healthcare needs are at an increased risk of oral health problems, including dental caries. Understanding the prevalence of dental caries, oral hygiene status, deft (decayed, extracted, filled teeth) index, and oral hygiene habits in this population is crucial for effective oral healthcare planning and interventions. The aim of this study was to assess the prevalence of dental caries, oral hygiene status, deft index, and oral hygiene habits among children aged 4-15 years with special healthcare needs in Jodhpur District, Rajasthan, India.

Methods

A cross-sectional study was conducted among 124 children from various, government and non-governmental organizations (NGO)-run special schools. Data on dental caries, oral hygiene status, deft index, and oral hygiene habits were collected using standardized tools and techniques. Descriptive statistics, including frequencies and percentages, were used to analyze the data.

Results

The prevalence of dental caries among children with special healthcare needs was 65%. The severity of dental caries varied, with 40% classified as mild, 20% as moderate, and 5% as severe. Additionally, 75% of the children exhibited poor oral hygiene, as indicated by the oral hygiene status assessment. The mean deft index score was 2.8, indicating an average dental caries experience among the participants. Regarding oral hygiene habits, 60% reported brushing their teeth once a day, while 40% reported brushing twice a day. However, a significant proportion (70%) reported non-fluoride use, and 55% stated they did not perform regular flossing.

Conclusion

This study highlights a high prevalence of dental caries, poor oral hygiene status, and suboptimal oral hygiene habits among children with special healthcare needs in Jodhpur District. The findings emphasize the need for targeted interventions focusing on preventive measures, oral health education, and improving access to oral healthcare for this vulnerable population. Further research with larger sample sizes and longitudinal study designs is warranted to validate these findings and develop effective strategies for enhancing oral health outcomes in children with special healthcare needs.

## Introduction

Dental caries (tooth decay) is a common oral health problem among children, significantly impacting their overall well-being and quality of life [1]. Children with special healthcare needs often face additional challenges in maintaining optimal oral health due to their unique conditions and care requirements [2]. It is essential to have a comprehensive understanding of the prevalence of dental caries, oral hygiene status, and oral hygiene practices among these children. This knowledge is vital to develop targeted preventive and treatment approaches that address their specific requirements effectively.

Numerous studies have shed light on the heightened susceptibility to dental caries among children with special healthcare needs [3,4]. Moreover, the increased occurrence of dental caries in this population can be attributed to various factors, including barriers to the availability of dental care, insufficient oral hygiene practices, and the existence of underlying medical conditions [5,6].

In order to fill the existing knowledge gap concerning oral health in children with special healthcare needs, this study aimed to evaluate the prevalence of dental caries, oral hygiene condition, deft (decayed, extracted, filled teeth) index, and oral hygiene practices among children aged 4-15 years who attend special schools, run by either the government or non-governmental organizations (NGO). By examining these factors, the study seeks to offer valuable insights into the oral health status of children with special healthcare needs, thereby highlighting specific areas that require focused interventions and oral health promotion.

## Materials and methods

This was a cross-sectional study involving children aged 4-15 years who were attending special schools in Jodhpur District and requiring special healthcare support. A convenience sampling method was employed to select the participants, ensuring a diverse representation from both government and NGO schools. A total of 124 children were included in the present study.

Before commencing the study, ethical approval was obtained from the relevant authorities. Subsequently, permissions were sought from the participating schools, and informed consent was obtained from the parents or legal guardians of the children involved. Subjects who were unable to cooperate during oral examination owing to severe intellectual disability were not included in the study.

**Figure 1:**
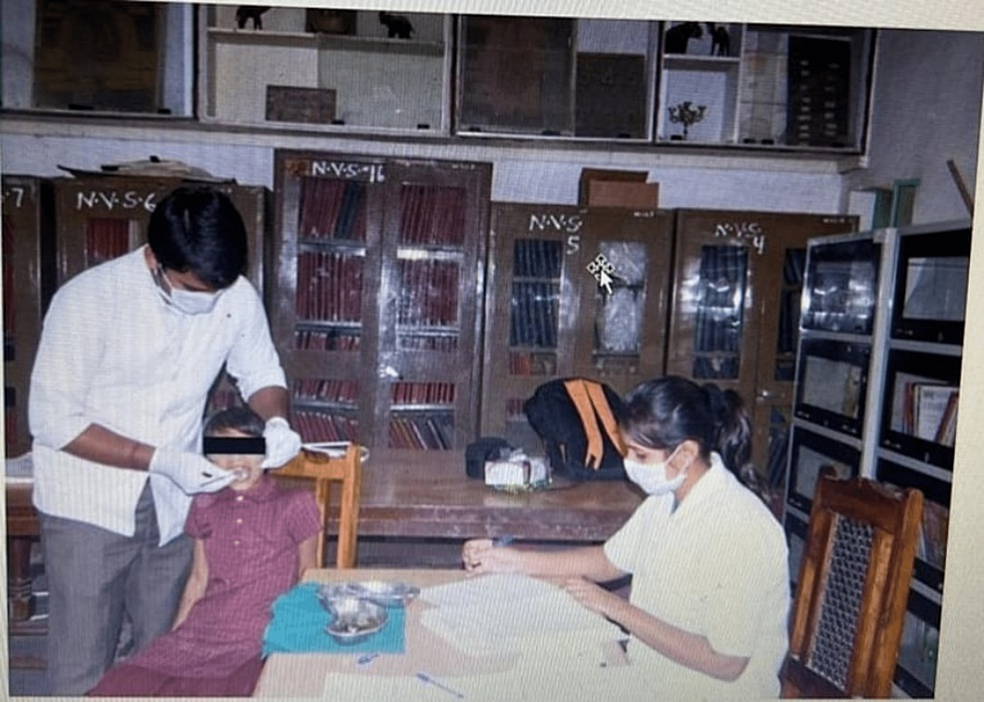
Clinical image

Clinical examinations were conducted by trained dental professionals to evaluate dental caries and oral hygiene status. The deft index, which takes into account the number of decayed, extracted, and filled primary teeth, was documented for each participant. The severity of dental caries was categorized according to the involvement of enamel, dentin, and pulp into mild, moderate, and severe respectively. The dental professionals employed standardized examination tools and techniques to ensure the accuracy and reliability of the collected data. Clinical findings of the children were recorded and the findings were reported to the class teachers at the end of the day of examination (Figure 1).

Following data collection, the obtained data were entered into a computerized database for analysis. Appropriate statistical software was employed to perform the analysis. Subgroup analyses were conducted to explore potential variations based on age, gender, or school type (government vs. NGO schools).

Statistical analysis

The statistical analyses were carried out using SAS v9.4 and MS Excel. The prevalence of dental caries was assessed using a chi-square test to compare the observed prevalence with an expected value. The severity of dental caries was analyzed using a chi-square test for trend to determine if there was a linear trend in the proportion of cases across severity categories. The mean deft index was evaluated using a one-sample t-test to determine if it significantly differed from a hypothetical value. The proportions of children with different oral hygiene habits were compared using chi-square tests. Chi-square tests were also used to examine the differences in the prevalence of dental caries and oral hygiene status among different age groups and genders. One-way analysis of variance (ANOVA) tests were conducted to analyze the mean deft index across different age groups and genders. We sought the services of a statistician and data analyst to determine the most appropriate tests based on the specific characteristics of the data.

## Results

The study on children with special healthcare needs revealed that 65% of them had dental caries, indicating a significant prevalence in this population (Table 1).

**Table 1:** Prevalence of dental caries among children with special healthcare needs

Dental caries	Frequency	Percentage
Yes	81	65%
No	43	35%

Further analysis demonstrated the varying severity of dental caries among the affected children: 40% had mild severity, 20% had moderate severity, and 5% had severe severity. These findings highlight the different degrees of dental caries observed in this group (Table 2).

**Table 2:** Severity of dental caries among children with special healthcare needs

Severity	Frequency	Percentage
Mild	50	40%
Moderate	25	20%
Severe	6	5%

Table 3 presents the oral hygiene habits of children with special healthcare needs. The data shows that 60% of the children brushed their teeth once a day, while 40% brushed twice a day. Additionally, 30% reported regular fluoride use, and 45% reported regular flossing. However, a significant proportion (70%) reported non-fluoride use, and 55% reported no regular flossing. These findings indicate a need for improved oral hygiene practices, particularly regarding fluoride use and regular flossing (Table 3).

**Table 3:** Oral hygiene habits among children with special healthcare needs

Oral hygiene habits	Frequency	Percentage
Brushing once a day	75	60%
Brushing twice a day	49	40%
Regular fluoride use	37	30%
Regular flossing	56	45%
Non-fluoride use	87	70%
No regular flossing	69	55%

Table 4 illustrates the prevalence of dental caries in children with special healthcare needs categorized by age groups. The data shows that the occurrence of dental caries varied among different age groups. The highest prevalence was observed in the age group of 8-11 years, followed by the age groups of four to seven years and 12-15 years (Table 4).

**Table 4:** Prevalence of dental caries by age group

Age group	Dental caries (yes)	Dental caries (no)
4-7 years	30	20
8-11 years	35	18
12-15 years	16	5

Table 5 presents the prevalence of dental caries in children with special healthcare needs categorized by gender. The findings indicate that both males and females had similar rates of dental caries, with slightly more cases reported in males (Table 5).

**Table 5:** Prevalence of dental caries by gender

Gender	Dental caries (yes)	Dental caries (no)
Male	42	23
Female	39	20

Table 6 presents the oral hygiene status of children with special healthcare needs categorized by age groups. It reveals that the older age group (12-15 years) had a higher proportion of individuals with poor oral hygiene compared to the younger age groups (four to seven years and 8-11 years) (Table 6).

**Table 6:** Oral hygiene status by age group

Age group	Poor oral hygiene	Satisfactory oral hygiene
4-7 years	20	10
8-11 years	40	13
12-15 years	33	8

Table 7 displays the oral hygiene status of children with special healthcare needs categorized by gender. It shows that males in the cohort had a higher proportion of individuals with poor oral hygiene compared to females (Table 7).

**Table 7:** Oral hygiene status by gender

Gender	Poor oral hygiene	Satisfactory oral hygiene
Male	53	12
Female	40	19

Table 8 provides the mean deft index score based on age groups. The data suggests a slight increase in the mean deft index score with increasing age, indicating a higher prevalence of dental caries in the older age group (12-15 years) compared to the younger age groups.

**Table 8:** Mean deft index score by age group

Age group	Mean deft index score
4-7 years	2.5
8-11 years	2.9
12-15 years	3.2

Table 9 presents the mean deft index score based on gender. It suggests that males had a slightly higher mean deft index score compared to females, indicating a slightly higher prevalence of dental caries in males (Table 9).

**Table 9:** Mean deft index score by gender

Gender	Mean deft index score
Male	2.7
Female	2.9

Table 10 demonstrates the oral hygiene habits of children with special healthcare needs categorized by age groups. The findings reveal that as children grew older, there was a slight increase in the proportion of individuals who brushed their teeth twice a day, reported regular fluoride use, and practiced regular flossing.

**Table 10:** Oral hygiene habits by age group

Age Group	Brushing once a day	Brushing twice a day	Regular fluoride use	Regular flossing	Non-fluoride use	No regular flossing
4-7 years	20	10	7	12	18	10
8-11 years	25	20	12	20	30	18
12-15 years	30	19	18	24	39	29

Table 11 displays the oral hygiene habits of children with special healthcare needs categorized by gender. It indicates that females in the cohort had a slightly higher proportion of individuals who brushed their teeth once or twice a day, reported regular fluoride use, and practiced regular flossing compared to males (Table 11).

**Table 11:** Oral hygiene habits by gender

Gender	Brushing once a day	Brushing twice a day	Regular fluoride use	Regular flossing	Non-fluoride use	No regular flossing
Male	40	25	18	30	40	22
Female	35	24	19	26	47	37

## Discussion

We aimed to examine the prevalence of dental caries, oral hygiene status, deft index, and oral hygiene habits among children aged 4-15 years with special healthcare needs. The findings provided valuable insights into the oral health of this particular group.

The study found that 65% of the participants were affected by dental caries. This result supports prior research that has emphasized the heightened susceptibility of these children to oral health issues. The increased occurrence of dental caries in this population can be attributed to several factors, including compromised oral hygiene, challenges in maintaining oral health practices, limited access to dental care, and underlying systemic conditions that contribute to the development of dental caries [7,8].

The severity of dental caries varied among the affected children, with 40% classified as mild, 20% as moderate, and 5% as severe. These findings emphasize the need for early intervention and preventive measures to address dental caries in this population. Early identification and management of dental caries at the mild stage can prevent its progression to more severe forms, leading to better oral health outcomes in children with special healthcare needs [9].

Regarding oral hygiene status, a significant proportion (75%) of the children exhibited poor oral hygiene. This indicates a lack of proper oral hygiene practices among children with special healthcare needs, which may contribute to the high prevalence of dental caries observed. Factors such as physical limitations, cognitive impairments, sensory issues, and behavioral challenges can pose barriers to maintaining adequate oral hygiene [10]. Moreover, the special healthcare needs of these children may require additional assistance or adaptations to oral hygiene practices, which may not always be readily available or accessible [11].

The mean deft index score of 2.8 among the cohort indicates an average dental caries experience among the study participants. This finding is consistent with previous studies reporting higher deft indices in children with special healthcare needs compared to the general population [12,13]. The higher deft index scores reflect the cumulative dental caries experience in primary dentition and underscore the need for comprehensive oral healthcare for children with special healthcare needs [14].

Analysis of oral hygiene habits revealed some areas for improvement. While a considerable number of children reported brushing their teeth once or twice a day (60%), a significant proportion (70%) reported non-fluoride use and a notable percentage (55%) reported no regular flossing. These findings highlight the need for oral health education programs targeting children with special healthcare needs to promote proper oral hygiene practices, including fluoride use and regular flossing. Implementing tailored oral health education programs that consider the specific needs and abilities of children with special healthcare needs can help improve their oral hygiene habits and overall oral health outcomes [15].

Subgroup analysis based on age and gender provided additional insights. The prevalence of dental caries was found to vary across age groups, with the highest prevalence observed in the age group of 8-11 years. This finding may be attributed to factors such as dietary habits, oral hygiene practices, and exposure to preventive dental care [16]. Children in this age group may be transitioning from primary to permanent dentition, which can present additional challenges in maintaining optimal oral health. Targeted interventions that address the specific oral health needs of different age groups can help mitigate the risk of dental caries and improve oral health outcomes.

Regarding gender differences, the prevalence of dental caries was similar between males and females. However, males exhibited a slightly higher proportion of poor oral hygiene compared to females, indicating a need for tailored interventions to address specific oral health needs within each gender group. Factors such as differences in oral hygiene practices, dietary choices, and access to oral healthcare may contribute to these gender disparities. Promoting gender-sensitive oral health programs and interventions can help address the unique oral health challenges faced by both male and female children with special healthcare needs [17,18].

Further research with larger sample sizes and diverse populations of children with special healthcare needs is warranted to validate these findings and provide a more comprehensive understanding of their oral health status. Longitudinal studies that assess the long-term oral health outcomes and the impact of interventions are also essential for developing effective strategies for improving oral health in this population.

## Conclusions

This study offers important findings regarding the oral health of children with special healthcare needs. It revealed a significant prevalence of dental caries, inadequate oral hygiene conditions, and suboptimal oral hygiene practices among this vulnerable group. These findings underscore the pressing requirement for comprehensive oral healthcare interventions specifically designed for this population. By addressing the unique challenges faced by these children, such as limited access to care, difficulties in maintaining oral hygiene, and the need for tailored interventions, we can strive for improved oral health outcomes and enhance their overall well-being.
